# Academic boredom(s): a person-centered investigation

**DOI:** 10.3389/fsoc.2023.1190872

**Published:** 2023-08-04

**Authors:** Dirk Tempelaar, Alexandra Niculescu

**Affiliations:** ^1^Department of Quantitative Economics, School of Business and Economics, Maastricht University, Maastricht, Netherlands; ^2^Centre for Learning Science, École Polytechnique Fédérale de Lausanne, Lausanne, Switzerland

**Keywords:** academic boredom, activity boredom, epistemic boredom, person-centered modeling, dispositional learning analytics, boredom antecedents

## Abstract

Should we refer to boredom or boredoms? Research on the emotion of boredom sets itself apart from studies on other emotions by posing the question: is boredom a singular concept or does it have multiple facets? In this manuscript presenting empirical research on academic boredom, our aim is to demonstrate the justification for claiming a distinct position. Person-centered models examining university students' achievement emotions reveal the existence of multiple types of boredom, in contrast to other learning emotions that are typically represented as singular constructs. Using data generated by dispositional learning analytics applications, we further investigate the progression of learning boredom over time, exploring the impact of the pandemic and analyzing how various student learning aptitudes, such as mindsets, epistemological beliefs, epistemic emotions, learning motivation, engagement, as well as demographic factors like gender and culture, can be considered as potential antecedents or triggers of boredom. Consistent with the control-value theory of achievement emotions, we conclude that control and value constructs serve as proximal antecedents of boredom, alongside epistemic boredom as a distal antecedent. However, the relationships between boredom and its antecedents exhibit notable variations across different types of boredom.

## 1. Introduction

“If you are immune to boredom there is literally nothing you can't accomplish” (Wallace, 2011; p. 440). But most of us are not immune to boredom. Everyone will be touched by boredom at least once in their life or experience it from time to time. The question is however, in what way, how frequently and how intense? The answer to this question will determine the implications or consequences that will come from the experience of boredom.

While research warns against the negative impact of boredom on health-related quality of life already in young adults (Schwartze et al., 2021), it is highly acknowledged that boredom continues to be experienced through the life spam and expand in different areas and environments: personal, at work and any other learning and achievement related situations. Furthermore, Wallace (2011) argues that the figure of boredom has become a villain in today's western society, obsessed by productivity, success and fear of failure. Although boredom is a recurrent emotion encountered in all aspects of life, it remains misinterpreted. From the highly-functioning professional who can hide symptoms of depression under an apparent emotional and mental disengagement, to the individual who seemed to have lost interest in daily activities but in fact lacks a sense of meaning in life and is severely depressed, boredom is an emotion that can strongly relate or even overlap with serious negative aspects of quality of life (Gerritsen et al., 2015). However, if explored enough, boredom can be the pathway to wellbeing and, when endured enough, lead to self-discovery. This requires an understanding of what boredom exactly is and at the same time of what is not, what are the factors that determine it and the conditions under which one can self-regulate and achieve more positive states in order to rich greater potential in life.

Maybe one of the most relevant settings to study and understand boredom is the achievement context: where individuals perform toward a goal and therefore are involved in a learning process that can be observed and influenced by both the learners themselves as well as by the environment. Especially in educational settings, the study of boredom remains a subject still not sufficiently investigated among adult learners (Loukidou et al., 2009). Beyond how often boredom is encountered and how intense it is felt, recent educational studies (Nett et al., 2011; Goetz et al., 2014; Tempelaar and Niculescu, 2022) address the issue of how exactly it is experienced: what makes the experience of boredom different for various individuals or, put differently, are there different types of boredom? The first step in answering this question starts with how we conceptualize the notion of learning boredom, an inescapable emotion, and how we observe it.

The metaphor used to describe boredom's role in educational studies is not often that of the elephant in the room but typically that of the silent emotion (Goetz et al., 2014). That metaphor still acknowledges the existence of an obvious problem: in early work on the role of emotions in educational achievement settings (Pekrun et al., 2010), boredom is recognized as one of the most frequent emotions. However, the metaphor of the silent emotion emphasizes another facet of boredom: the difficulty of observing it. Or to use another characteristic for that same facet: the invisibility of boredom (Finkielsztein, 2021). Avoiding to discuss the elephant's presence may be partially explained by the methodological difficulty of collecting boredom measurements, forcing researchers to use indirect measures. “… it is much more productive to collect descriptions and explanations of boredom in hindsight. This is why most studies in the field rely mainly on verbal expressions rather than on observations *in vivo*.” Concludes Finkielsztein (2021, p. 18) regarding an alternative source of boredom data. Therefore, in order to properly capture the experience of boredom, the choice of a method though which the concept is operationalized and assessed validly is key.

### 1.1. Aim of the study

In this study, we will follow that same route by using self-reported boredom measures using verbal expressions of first-year university students in a challenging achievement setting. It is well-known that “few people are capable of gaining insight into their own emotions” (Finkielsztein, 2021, p. 18). However, in achievement settings, individuals' self-perceptions of their abilities play a crucial role, despite the presence of bias in these self-perceptions, which contains valuable information (Tempelaar et al., 2020). Boredom arises at the crossroads of external circumstances and personality-related issues (Ros Velasco, 2019). Given the context of our study, which examines a highly diverse sample of students operating within the same external environment, we can specifically focus on the role of personality and explore the extent to which “boredom is in your mind” (Ros Velasco, 2019).

In the remainder of this introductory section, we will first clarify the concept of boredom and place it in relation to existing learning theories, in particular Self-Regulation and Flow Theory, in order to show its impact on performance; we then discuss a classification of boredom types and models and lastly, explain academic and epistemic boredom as achievement emotions within the framework of the Control and Value Theory of Achievement Emotions The research questions are presented at the end of this section. Defining boredom and placing it among emotion theories.

While there is no agreement on a specific definition of boredom (Finkielsztein, 2021), we choose to view it as the personal encounter with a situation that is often undesired and unpleasant (Eastwood et al., 2012). It is characterized by a sense of time passing slowly, disengagement on both behavioral and cognitive levels (Goetz and Hall, 2014), and a challenge in maintaining focus (Elpidorou, 2018), leading to a desire to escape the current moment (Westgate and Wilson, 2018). From this perspective, boredom in this study is seen as a state emotion.

As a state emotion more generally, boredom can be characterized along three main features. First, it is a person generated response within a particular situation that is directed to a relevant goal (Scherer et al., 2001). Second, it “coordinates the responses to that particular situation that generated it” (Jacobs and Gross, 2014, p. 184). For instance, a person can start to withdraw from a boring situation depending on the intensity of the emotion experienced. In other words, such response can serve an adaptive or maladaptive function, depending on how the situation is perceived and the immediate goal of the person involved. Third, the activation response of the emotion is characterized by a “degree of malleability” (Jacobs and Gross, 2014, p. 185): the behavior induced by the emotion of boredom is under the volitional control of the person. This is the core point that makes possible for the emotion to be regulated. Consequently, the regulation of an emotion also implies that its functional aspect can be changed by the awareness of the individual: a person can decide to remain in, instead of leaving from, what is perceived as a boring situation by (re)adjusting their appraisals and therefore, (re)calibrating either their level of interest, the intensity of the emotional experience or the goal of a particular activity. In this sense, *emotional regulation* refers to “the processes by which individuals influence which emotions they have, when they have them, and they experience and express these emotions” (Gross, 1998, p. 275). Therefore, the regulation of emotion responses by specific strategies is crucial, as it determines the success or failure of various situations. From this point of view, it can be argued that boredom is a crucial emotion in educational contexts as, generally speaking, it tends to have a maladaptive function for achievement, where deregulatory strategies are more often deployed which, in turn, disrupt the process of learning.

Since in study settings, the ideal remains to create optimal experiences and conditions during the learning process by moving beyond maladaptive or deregulatory behaviors, cognitions and emotional states, an understanding of boredom in such contexts is of central importance (Csikszentmihalyi, 2000). An optimal experience, or flow, is according to Csikszentmihalyi (2000) a state of intense focus, a perceived fast passage of time and engagement with an activity that leads to improved performance. In this regard, it can be considered that the experience of flow places itself at the other side of the spectrum when compared to boredom and its implications for learning. Therefore, understanding the conditions that favor the experience of flow are equally relevant for addressing the malfunctions of boredom. Research into flow (Egbert, 2003), has described a number of conditions that are present during such experiences: (a) a perceived balance of skills and challenge, (b) opportunities for intense concentration, (c) clear task goals, (d) feedback that one is succeeding at the task, (e) a sense of perceived control, (f) a lack of self-consciousness, and (g) a perceived fast passage of time. The first condition referring to the learners' perceived skills and being challenged, appears most relevant for the conceptualization of boredom, in the sense of how boredom as an emotion develops from its antecedents; indeed, Whalen (1997) points that it is exactly the balance between the two factors that allows for the condition of flow to occur: high challenge with a high self-perception of skills create an optimal balance for flow, while a high challenge and low skills can create anxiety, and low challenge and high skills favor boredom. More recently, for the boredom case, it was argued that is not only a context of low challenge but also of too high challenge that can induce this emotion (Pekrun et al., 2010). This implies that the experience of boredom in its mal-adaptive expression is, in fact, a detrimental condition for reaching flow and therefore lowering the chances of improved performance.

### 1.2. Classifying boredom according to different boredom models

Westgate and Wilson (2018) categorize models explaining state boredom into three groups. The first group comprises environmental theories that attribute boredom to the external context, focusing on insufficient external stimulation (Goetz et al., 2006, 2014) or the impact of external constraints (Pekrun, 2006; Goetz et al., 2014). The second group consists of attentional theories that highlight the role of internal psychological processes, suggesting that boredom stems from attentional failures (Eastwood et al., 2012). The third group encompasses functional theories of boredom, which examine its underlying purpose in terms of goals, opportunity cost, or finding meaning. According to this perspective, boredom is considered a functional emotion as it signals a need for change and serves a regulatory role that can potentially enhance an individual's wellbeing (Elpidorou, 2018).

In addition to the three aforementioned sets of models, Westgate and Wilson (2018) introduce a fourth model called the Meaning and Attention Components (MAC) Model of Boredom. The MAC Model incorporates the previous perspectives on boredom by suggesting that individuals experience this emotion when they are either “unable or unwilling” (Westgate and Wilson, 2018, p. 693) to engage with a particular task. As a result, the MAC Model proposes that different types of boredom can arise depending on the triggers of boredom, such as the lack of meaning or attention deficit. However, it's important to note that ongoing research exploring these models still deliberates on one aspect of boredom: its arousal or activation dimension.

Research findings have indicated that state boredom can be defined in terms of low arousal, high arousal, or a combination of both, encompassing varying levels of arousal flexibility (Eastwood et al., 2012; Goetz et al., 2014; Elpidorou, 2020). To account for these conflicting explanations, Goetz and Hall (2014) propose two possible interpretations. Firstly, they argue that the concept of arousal itself might be multidimensional. Secondly, they suggest the existence of multiple types of boredom. While some authors have contested the latter possibility based on associated arousal levels (Elpidorou, 2020), the issue of heterogeneity in defining boredom remains a long-standing debate (Goetz et al., 2014; Westgate and Wilson, 2018; Elpidorou, 2020).

Beyond state boredom, as presented in the models described above, which is limited to a particular situation and therefore also labeled as situational, boredom studies typically distinguish state and trait attributes of boredom; see, e.g., Sharp et al. (2021) and Bambrah et al. (2023). Finkielsztein (2021) pleads for a different but related typology: situational, chronic, and existential boredom. Situational boredom results from the interplay of personal aptitudes and the external context and is clearly a state. Chronic boredom follows from recurrent states best corresponds with the trait attribute of boredom; this is an intermediate kind of boredom that results from an “accumulation of situational boredoms due to its frequent repetition in similar circumstances” (Finkielsztein, 2021, p. 79). This contrasts mainstream psychology research that applies trait boredom or boredom that has evolved into a personality characteristic as an intermediate kind. At last, existential boredom is more a mood than an emotion. Compared to situational boredom as a state emotion, existential boredom can be found at the other pole on the continuum, the omnipresent boredom version (Finkielsztein, 2021). In this research, we aim to combine these views by using different sub-sets of academic emotions (Pekrun and Linnenbrink-Garcia, 2012) which will introduce in the next section.

### 1.3. Academic boredom and epistemic boredom as achievement emotions

Boredom encountered within an academic environment is commonly referred to as academic boredom (Pekrun et al., 2011). This emotional state can manifest in various situations within academic settings, including attending lectures or classes (class boredom), taking exams (test boredom), and engaging in activities like homework, studying, or other learning tasks (learning boredom). According to this conceptualization, boredom is regarded as a situational experience linked to a specific learning activity or outcome (Pekrun et al., 2010). In concluding the conceptualization of boredom, it is important to address its relationship with other emotions within the same learning context. Prior research indicates that academic boredom exhibits positive associations with other negative emotions and negative associations with positive emotions (Goetz et al., 2006; Pekrun et al., 2011; Niculescu et al., 2015). The moderate strength of these relationships suggests that boredom is distinct from other negative emotions and does not solely represent the absence of positive emotions (Goetz and Hall, 2014).

Furthermore, when students encounter learning tasks that elicit similar emotional responses, these emotions are classified as epistemic academic emotions. Epistemic emotions pertain to cognitive aspects of the task, with curiosity, and confusion being prototypical examples (Pekrun and Linnenbrink-Garcia, 2012; Zheng et al., 2023). Epistemic emotions share trait-like characteristics and exhibit essential features akin to chronic emotions, as defined by Finkielsztein (2021). More precisely, while the achievement-related emotions focus on the experience of engaging in a learning activity, epistemic emotions pertain to the cognitive aspects of the task itself (Pekrun and Linnenbrink-Garcia, 2012). Epistemic boredom is the boredom experienced with the process of learning, understanding, and knowledge acquisition. This boredom type reflects individuals' perceptions of their own understanding, the effort they invest, and the progress they make in gaining knowledge or solving problems. In this research, we use epistemic emotions as an antecedent of academic boredom.

The Control-Value Theory of Achievement Emotions (CVTAE; Pekrun, 2006) presents a framework that examines the factors leading to and resulting from achievement emotions in relation to academic activities and outcomes. According to the CVTAE, achievement emotions are characterized along two dimensions: valence and arousal. In the case of boredom, it is characterized by negative valence and deactivating arousal (Pekrun et al., 2010). Boredom arises from beliefs about having low control over outcomes, which are referred to as control appraisals (Pekrun, 2006; Frenzel et al., 2007). Control and value serve as immediate antecedents of boredom and other achievement emotions, and they are influenced by distal antecedents related to both individual characteristics and the learning environment (Pekrun, 2006). Gender and achievement goal setting are examples of distal antecedents within the individual characteristics category (Pekrun, 2006).

The majority of studies examining achievement emotions and their antecedents measure emotions based on the intensity of emotional sentiment or the degree of boredom experienced. In studies investigating different types of boredom, Goetz et al. (2014, 2016) utilized person-oriented modeling approaches to analyze the frequency of boredom experiences, resulting in the identification of five distinct types of boredom. The first type, called “indifferent” boredom, was characterized by low arousal and a relatively positive valence. The second type, “calibrating” boredom, exhibited higher arousal than indifferent boredom and a somewhat negative valence. The third type, “searching” boredom, displayed higher arousal and more negative valence compared to calibrating boredom. The fourth type, “reactant” boredom, demonstrated the highest levels of arousal and negative valence among all the types. Lastly, the fifth type, an unexpected finding in Goetz et al. (2014) study, was labeled as “apathetic” boredom, distinguished by a low level of arousal and a strongly negative valence. Importantly, the first four types of boredom exhibited a linear relationship along the dimensions of valence and arousal, while apathetic boredom fell outside of this linear pattern. This conceptualization of boredom suggests that it is not a singular construct and that its emotional experience encompasses various facets. Additionally, the different experiences of boredom appear to be influenced by the underlying factors contributing to the emotion (Westgate and Wilson, 2018).

As stated by Krannich et al. (2019, p. 208), the primary factors leading to boredom are individuals experiencing states of being over- or under-challenged (Finkielsztein, 2021 also supports this view). This explanation aligns with the Control-Value Theory of Achievement Emotions (CVTAE), where boredom arises from a combination of a perceived lack of control and a lack of value. This occurs when the learning task is perceived as unimportant and either too demanding or insufficiently challenging (Pekrun et al., 2010).

### 1.4. Research questions

In addition to the overarching research question regarding learner aptitudes as antecedents of boredom, we aim to delve deeper into the impact of the pandemic by exploring the following questions: Can we detect any changes in the levels of activity and epistemic boredom over time? Moreover, do gender differences emerge in the developmental patterns of these two boredom emotions? Significantly, we seek to determine whether boredom should be considered as a singular concept or if it can be categorized into different types. Lastly, we are keen on examining the associations between profile characteristics and potential antecedents of boredom.

## 2. Methods

In the field of educational research, investigations into hypothesized relationships often rely on variable-centered modeling techniques like regression, factor analysis, or structural equation modeling. These methods are employed to analyze the inter-individual variations within the entire sample, as demonstrated by studies conducted by Howard and Hoffman (2018), Zhang (2022), and Bambrah et al. (2023). It is important to note that this approach operates under the assumption of a homogeneous sample. When homogeneity is lacking, person-centered modeling approaches (Howard and Hoffman, 2018; Sharp et al., 2021) aim to group individuals within each category, similar and different from individuals in other categories. Given that research questions are commonly formulated at the variable level, addressing heterogeneity requires a two-step approach. The first step involves employing a person-centered approach to partition a heterogeneous sample into more homogeneous clusters or classes. The subsequent step entails utilizing a variable-centered approach on the resulting homogeneous sub-samples. In our study, we utilized K-means Cluster Analysis in SPSS to decompose the heterogeneous sample into these more homogeneous subsamples.

### 2.1. Participants and educational context

The participants in this study consisted of first-year students enrolled in a business and economics school located in the southern region of the Netherlands. These students were part of fourteen recent cohorts, spanning from the academic year 2010/2011 to 2022/2023. The study particularly focused on the four most recent cohorts, ranging from 2019/2020 to 2022/2023. The educational programs offered by this school differed from mainstream European university education in two key aspects: they employed student-centered instruction through problem-based learning (PBL) and the English-language programme had a strong emphasis on internationalization, attracting a significant number of international students.

Among the 5,296 first-year students included in the cluster analysis, 38% were female, and 62% were male. The sample consisted of students from 95 different countries, reflecting the diverse educational backgrounds. This information was utilized to create culture dimension scores and region indicator variables. It is worth noting that most students enrolled in the program immediately after completing their high school education, with the typical age range falling between 18 and 20 years old. The data were collected during the introductory module on mathematics and statistics, which served as the first module of the program.

Among the four most recent cohorts, there was one pre-COVID-19 cohort, two cohorts that were affected by COVID-19, and one post-COVID-19 cohort. The impact of COVID-19 on education was more significant in the 1^st^ year of the pandemic compared to the second. In the academic year 2020/2021, all lectures were transitioned to online format, and small-group tutorial meetings were conducted via Zoom. In the following year, 2021/2022, lectures remained online, but tutorial meetings had a mixed approach, with most being held on campus and some conducted through Zoom. The final cohort included in this study, the 2022/2023 cohort, represents a post-COVID-19 period where university education fully resumed on campus for all contacts. However, these students had experienced 2 years of high school education during the COVID-19 pandemic.

Undoubtedly, COVID-19 brought about the most significant changes in the educational system and its context throughout the considered period. It is worth noting that our module was an early adopter of technology-enhanced education, and over the span of the fourteen cohorts included in the analysis, both the technological aspect and the problem-based learning tutorials remained relatively stable.

The educational backgrounds of students vary based on their nationality and mathematical track. In many national educational systems, there are three distinct tracks that prepare students for arts and humanities, social sciences, and sciences. For our programs, the second level of mathematical track is a requirement, but interestingly, 37% of students had received mathematical education at the highest level, denoted as MathMajor.

The diversity in students' prior education and knowledge gives rise to a range of challenges that students encounter as they progress through the module. Consequently, it is expected that this diversity will lead to a variety of learning activity emotions experienced by the students.

Small tutorial groups consisting of 14 students are the setting for problem-based learning. To cater to the needs of students who may require additional support and to provide a diverse range of learning resources, online learning opportunities were introduced. This integration of online elements with traditional methods creates a blended learning environment. In the PBL tutorial sessions, the face-to-face component, the emphasis is placed on collaborative problem-solving, particularly for open-ended problems. This instructional approach, known as the “flipped classroom,” is discussed in detail by Williams et al. (2016).

Dispositional learning analytics is implemented to provide additional support for student learning. This approach, rooted in the field of learning analytics (Buckingham Shum and Deakin Crick, 2012), aims to offer students personalized feedback based on data obtained from their interactions with digital learning platforms. In the case of dispositional learning analytics (Rienties et al., 2019; Tempelaar et al., 2021), self-report surveys are used to gather information about students' learning dispositions, which serve as an additional data source. Learning dispositions encompass student aptitudes that are considered crucial personal factors influencing learning processes, according to social-cognitive learning theories.

The learning dispositions data used in this study originated from one of the module's student assessments, specifically the student project. The project involved the analysis of personal disposition data using statistical methods. To gather this data, students were instructed to administer various questionnaires focusing on the affective, behavioral, and cognitive aspects of their aptitudes during the 1^st^ week of the course. Several weeks later, they received their personal datasets for the project work.

It is worth noting that the survey administration for measuring aptitudes occurred early in the module, capturing dispositions developed during 6 years of high school education. The only exception to this timing was the administration of the activity emotions survey, which took place in the 4^th^ week of the module, precisely at the halfway point. This decision was based on the consideration that, by this time, students would be familiar with typical learning activities, while also ensuring that the final exam was distant enough to prevent the influence of test-related emotions on activity emotions.

In addition to capturing achievement emotions and epistemic learning emotions, the collected learning dispositions in this study include attitudes toward mathematics and statistics, measures of motivation and engagement, mindsets, beliefs about effort, goal-setting behaviors, academic motivations, and other learner data that are not utilized in this particular study. The selection of which dispositions to include in this study is guided by review studies like Finkielsztein (2021), which highlight the essential elements in defining boredom.

### 2.2. Materials

#### 2.2.1. Achievement and epistemic emotions

The Control-Value Theory of Achievement Emotions (CVTAE, Pekrun, 2006) proposes that achievement emotions vary in terms of their valence, focus, and activation. For this study, we utilized the Achievement Emotions Questionnaire (AEQ, Pekrun et al., 2011), an instrument grounded in the CVTAE framework, to select four learning activity emotion scales that are most strongly associated with academic performance from the total of eight activity scales included in the AEQ. These scales include positive activating *Enjoyment*, negative activating *Anxiety*, and negative deactivating *Boredom* and *Hopelessness*. Given the emphasis on independent, self-regulated learning within the PBL system, we specifically employed the learning activity-related versions of the scales, rather than the class- or test-related versions. This decision also influenced when the AEQ measurement took place: precisely at the midpoint of the module. At this specific moment, students have acquired enough familiarity with the regular learning activities, allowing them to express emotions related to those activities. Additionally, the timing ensures that the final exam is distant enough to prevent the blending of emotions experienced during the learning activities with those associated with the upcoming test. As the focus is on the learning activities themselves, the feeling of boredom during these activities is considered a temporary and situational form of boredom: a. boredom state.

To assess epistemic emotions, we employed the Epistemic Emotion Scales (EES, Pekrun et al., 2017), which encompass measures of *Surprise, Curiosity, Confusion, Anxiety, Frustration, Enjoyment*, and *Boredom*. The EES instrument was administered during the initial week of students' arrival at the university. As a result, the experience of epistemic boredom captured by this measurement reflects the cumulative boredom that students have encountered throughout 6 years of mathematics education in high school. In our study, this measurement represents the trait aspect.

As a key proximal antecedent of activity emotions, we included the measurement of *Academic Control*, utilizing the perceived Academic control scale developed by Perry et al. (2001). In this study, epistemic emotions are considered distal antecedents of achievement emotions, contributing to a comprehensive understanding of the emotional processes involved in academic activities.

#### 2.2.2. Culture

When examining cultural variations, our investigation adheres to the framework introduced by Hofstede et al. (2010). Hofstede identified six key dimensions that capture the differences between cultures: power distance, uncertainty avoidance, individualism-collectivism, masculinity-femininity, long-term-short-term orientation, and indulgence-restraint. *Power distance* pertains to the extent to which individuals within organizations and institutions accept and anticipate unequal distribution of power. *Uncertainty avoidance* reflects a society's inclination to tolerate ambiguity and uncertainty, indicating the level of discomfort experienced by its members in ambiguous situations. *Individualism* vs. *Collectivism* describes the degree to which individuals are integrated into groups, ranging from loose connections between individuals with an emphasis on self-reliance to strong bonds within cohesive in-groups. In *Masculine* societies, there is a distinct separation of emotional gender roles, while in *Feminine* societies, these roles tend to overlap. The fifth dimension, *Long-term* vs. *short term orientation*, distinguishes societies based on their orientation toward future rewards or the immediate fulfillment of present needs and desires. The sixth and most recent addition to the cultural dimensions is *Indulgence* vs. *Restraint*, which reflects the degree to which a culture permits or restricts the gratification of needs and human drives associated with hedonism and consumerism. For individual-level analysis, we utilize national scores on Hofstede's cultural dimensions to represent the cultural background of students who received secondary education in their respective countries. This approach aligns with the procedures followed by Hofstede et al. (2010), where cultural differences refer to variations between nations rather than variations within individuals. For further insights, refer to Tempelaar and Verhoeven (2016).

An alternative approach to operationalizing culture is presented by the GLOBE (Global Leadership and Organizational Effectiveness) research program, as outlined in the work of House et al. (2004). The GLOBE project aimed to identify and define nine cultural dimensions while also forming clusters of world cultures that transcend national boundaries. In our study, we have further refined the GLOBE culture clustering by categorizing cultural regions into Germanic Europe, Nordic Europe, Eastern Europe, Latin Europe, Anglo, Asia, Latin America, and Africa. To enhance the specificity of the Germanic Europe region, it has been subdivided into German-speaking countries, Netherlands, and Belgium. Additionally, separate regions were identified for countries that had over a hundred students within the four most recent cohorts, namely France, Spain, and Italy. For more information, refer to the works of Rienties and Tempelaar (2013) and Tempelaar et al. (2013).

#### 2.2.3. Attitudes toward learning

To augment the proximal control antecedent with the value component, we utilized an expanded version of the Survey of Attitudes Toward Statistics (SATS, Tempelaar et al., 2007). Drawing upon the expectancy-value theory (Wigfield and Eccles, 2000), this instrument encompasses six attitudinal dimensions related to learning quantitative methods. The instrument assesses *Affect* in learning quantitative topics, *Cognitive competence, Value*, expected difficulty in learning (reversed as *No Difficulty*), *Interest*, and planned *Effort*. Within these scales, we selected two scales to represent the extrinsic and intrinsic aspects of valuing mathematics and statistics learning: *Value*, which captures students' attitudes regarding the usefulness, relevance, and worth of the subject in their lives, and *Interest*, which measures the level of individual intrinsic interest.

#### 2.2.4. Motivation and engagement wheel measures

The Motivation and Engagement Survey (MES), which is based on the Motivation and Engagement Wheel framework (Martin, 2007), deconstructs learning cognitions and learning behaviors into four quadrants encompassing adaptive and maladaptive types as well as cognitive (motivational) and behavioral (engagement) types. Within the framework, the adaptive cognitive factors or positive motivations include *Self-Belief* , *Learning Focus*, and *Valuing School*. *Persistence, Task Management*, and *Planning* represent the adaptive behavioral factors or positive engagement. On the other hand, the maladaptive cognitive factors or negative motivations consist of *Uncertain Control, Failure Avoidance*, and *Anxiety*, while *Self*-*sabotage* and *Disengagement* represent the maladaptive behavioral factors or negative engagement.

#### 2.2.5. Mindset measures: self-theories of intelligence and effort-beliefs

Measures of self-theories of intelligence, encompassing both entity and incremental types, were adopted from Dweck's Theories of Intelligence Scale—Self Form for Adults (Dweck, 2006). This scale comprises eight items, consisting of four statements related to *Entity Theory* and four statements related to *Incremental Theory*. Measures of effort-beliefs were sourced from two references: Blackwell (2002) and Dweck (2006). Dweck's work presents sample statements that depict effort as both a negative concept (*Effort Negative*) and a positive concept (*Effort Positive*), highlighting the belief that exerting effort either conveys low ability or activates and enhances one's ability. Additionally, Blackwell (2002) comprehensive set of Effort beliefs was utilized, consisting of five positively phrased and five negatively worded items (see also Blackwell et al., 2007; Tempelaar et al., 2015). Goals were operationalized using the instrument developed by Grant and Dweck (2003), which differentiates between two mastery goals, namely *Challenge-Mastery* and *Learning Goals*, and four types of performance goals. Among the performance goals, two are associated with appearance, namely *Outcome* and *Ability Goals*, while the other two are normative in nature, namely *Normative Outcome* and *Normative Ability Goals*.

#### 2.2.6. Academic motivations

Vallerand et al. (1992) proposed three primary categories of motivations in learning: Intrinsic, Extrinsic, and Amotivation. Firstly, Intrinsic motivations are centered around the enjoyment and satisfaction derived from the task itself. Intrinsic motivation comprises three subcategories: *Intrinsic motivation to know*, which involves finding satisfaction in learning and understanding something new; *Intrinsic motivation toward accomplishments*, where individuals derive pleasure from achieving or creating something; and *Intrinsic motivation to experience stimulation*, which refers to the fulfillment gained from engaging in the activity. On the other hand, Extrinsic motivation encompasses a wide range of behaviors driven by external factors and pursued as a means to an end rather than for their inherent value. Extrinsic motivation can be further divided into three types: *External regulation*, which involves external rewards or constraints; *Introjection*, where individuals internalize the reasons behind their actions to some extent; and *Identification*, where the behavior is perceived as personally valuable and important. Finally, individuals are classified as *Amotivated* when they lack both intrinsic and extrinsic motivation, perceiving their actions as being controlled by external forces beyond their influence.

The study obtained ethics approval from the Ethical Review Committee Inner City faculties (ERCIC) of Maastricht University under file ERCIC_044_14_07_2017. Prior to participation, all individuals gave their informed consent for the utilization of anonymized student data in educational research.

### 2.3. Statistical analyses

This study applies a combination of person-centered and variable-centered statistical methods. The heterogeneity of the sample is a sufficient reason in itself to decompose the full sample into homogeneous sub-samples that satisfy the requirements of variable-centered methods: that of homogeneity of the data (Howard and Hoffman, 2018). However, substantive arguments to start the analysis with a person-centered approach add to statistical arguments. In the provision of learning feedback to students and the design of educational interventions, the main aim of applying learning analytics, it is attractive to seek common grounds rather than addressing every student on an individual basis (Tempelaar et al., 2021).

Person-centered modeling of activity emotions was performed with K-means cluster analysis based on all four activity emotions scores. In deciding upon the number of clusters, substantial criteria were applied (Howard and Hoffman, 2018).

In the second step of the analysis, we apply variable-centered statistical methods to investigate profile differences. These methods include ANOVA and *t*-tests for independent and paired samples. To restrict the accumulation of Type 1 error in performing multiple hypothesis tests, all tests are performed at the conservative significance level of 0.001 (*p* < 0.001). Large sample sizes generate statistical significant differences in most of these hypothesis tests, and therefore we opt to consider both statistical and practical significance, reporting effect sizes along *p*-values.

## 3. Results and discussion

### 3.1. Boredom types

**Figure 1:**
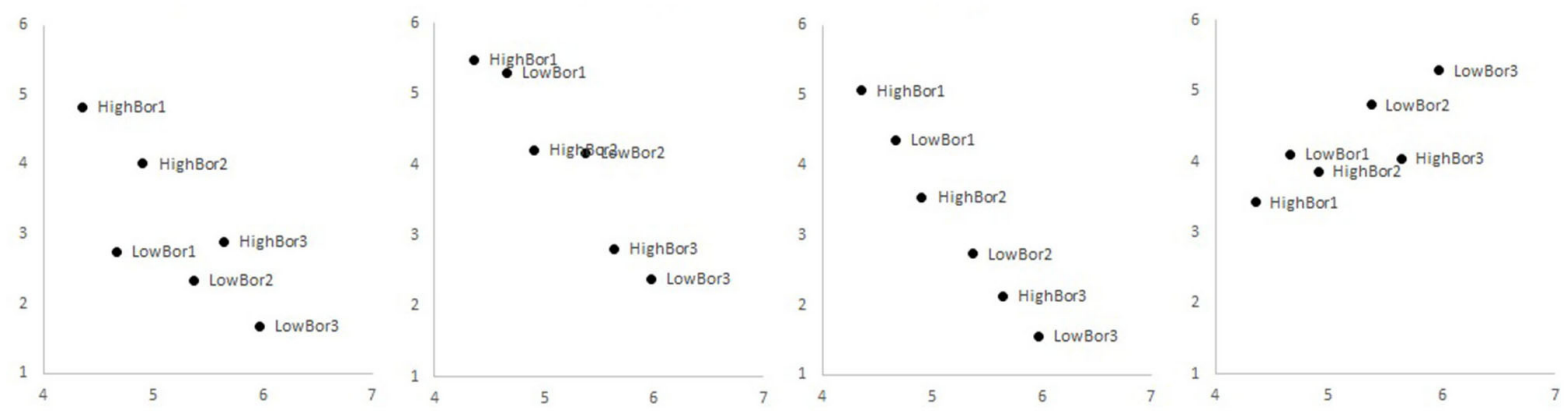
Activity emotion profiles in the plane of *Boredom, Anxiety, Hopelessness*, and *Enjoyment*, respectively, vs. *Academic Control*.

The creation of activity emotion profiles follows classification approaches applied by Goetz et al. (2014), Sharp et al. (2021), and Tempelaar and Niculescu (2022). Using data from the four most recent cohorts, cluster analyses based on *Anxiety, Boredom, Hopelessness*, and *Enjoyment* scores were run for a range of cluster numbers. All cluster solutions having four or more clusters demonstrate the existence of one or more high boredom profiles: clusters of students with extraordinarily high levels of boredom, relative to the levels of other learning emotions and levels of arousal. The cluster solution we opted for distinguishes six profiles, three of high boredom type and three of low boredom type. We will label these profiles as *HighBor1, HighBor2* and *HighBor3* for the high boredom clusters and *LowBor1, LowBor2* and *LowBor3* for the low boredom clusters. Figure 1 provides a graphical illustration of these profiles; Table 1 specifies mean levels. Control-value theory of achievement emotions predicts that levels of activity emotions are linearly related to *Academic Control*. Activity emotions with a negative valence are expected to be negatively related, and those with a positive valence are positively related, showing linear relationships. The third panel of Figure 1 confirms this expectation for the activity emotion *Hopelessness*: higher levels of *Academic Control* (horizontal axis) correspond to lower levels of *Hopelessness*, in a nearly exact negative linear relationship. Also, the second panel, sketching the relationship between *Academic Control* and *Anxiety*, complies with the theoretical framework: higher levels of *Academic Control* go with lower levels of *Anxiety* in a more or less linear manner. However, the first panel breaks this pattern: it suggests the existence of not one but two linear relationships, one for the high boredom profiles and a different one for the low boredom profiles. In both linear relationships, *Boredom* levels decrease with increasing *Academic Control* levels, but this relationship is at a higher level for the high boredom profiles. The fourth panel, that of *Enjoyment* vs. *Academic Control*, somewhat resembles the first panel; because *Enjoyment* is stronger correlated with *Boredom* than any other negatively valenced activity emotion, this does not come as a surprise.

**Table 1:** Descriptives of activity emotion profiles.

	***Academic Control***	***Activity Boredom***	***Activity Anxiety***	***Activity Hopelessness***	***Activity Enjoyment***	**Number of students**
*HighBor1*	4.35	4.82	5.48	5.06	3.43	423
*HighBor2*	4.91	4.01	4.21	3.54	3.86	952
*HighBor3*	5.64	2.89	2.80	2.12	4.05	777
*High Boredom*	5.06	3.76	3.95	3.33	3.84	2,152
*LowBor1*	4.66	2.74	5.30	4.35	4.10	695
*LowBor2*	5.37	2.34	4.16	2.73	4.80	1,049
*LowBor3*	5.98	1.67	2.38	1.55	5.29	699
*Low Boredom*	5.34	2.26	3.98	2.85	4.74	2,443
All students	5.21	2.96	3.97	3.08	4.32	4,595

### 3.2. Boredom in time: the impact of the pandemic

**Figure 2:**
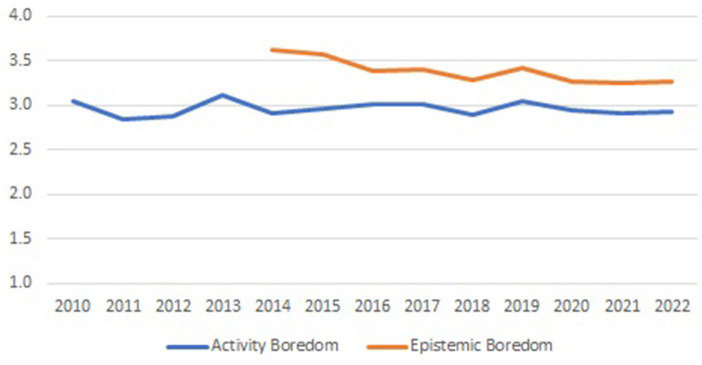
Development of *Activity* and *Epistemic Boredom* over time.

The potential impact of the pandemic is part of a more general question: can we observe any development in levels of epistemic and activity boredom over time, as in Ros Velasco (2023)? Making use of the full time span of boredom data (thirteen student cohorts, from 2010/2011 to 2022/2023 for *Activity Boredom*, nine student cohorts, from 2014/2015 to 2022/2023 for *Epistemic Boredom*), we observe a very stable progression over time (see Figure 2).

Beyond little variation over time, Figure 2 demonstrates that all boredom observations lay below four, the neutral value of the 1.0.7 Likert scale applied in measuring the activity and epistemic boredom items. Next, levels of activity boredom are consistently below levels of epistemic boredom. That difference comes with a large effect size: in a paired samples *t*-test, we find the effect size of the difference between *Epistemic* and *Activity Boredom* to be 31.5%.

Zooming into the last 4 years of observations, 2019/2020, the pre-pandemic year, 2020/2021 and 2021/2022 as the two pandemic years, and 2022/2023, the post-pandemic year, we observe in a two-way ANOVA with year and region as fixed factors, statistical significance for region, and lack of statistical significance for year, both regarding *Activity* and *Epistemic Boredom*. However, practical significance is very limited: with eta squared of 2.8 and 2.1% for region in activity and epistemic boredom, region explains <3% of total variation (eta squared is 0.2 and 0.5% for year in *Activity* and *Epistemic Boredom*, year explaining <1% of total variation).

### 3.3. Boredom and culture

Analyzing the influence of culture on boredom measures applying Hofstede's culture dimensions signals two dimensions being of statistical and practical significance: the *Masculinity* vs. *Femininity* dimension and the *Indulgence* vs. *Restraint* dimension (see Table 2).

**Table 2:** Correlations of boredom measures with Hofstede's national culture dimensions.

**Boredom**	***Power distance***	***Uncertainty avoidance***	***Individualism–Collectivism***	**Masculinity–Femininity**	***Long-term–Short-term orientation***	***Indulgence–Restraint***
Epistemic	−0.014	−0.040^**^	0.028	−0.095^***^	−0.018	0.132^***^
Activity	0.019	−0.029	0.055^***^	−0.125^***^	−0.037^*^	0.161^***^

^*^p < 0.05; ^**^p < 0.01; ^***^p < 0.001.

The *Masculinity* vs. *Femininity* dimension negatively correlates with boredom scores, indicating lower boredom levels in countries with a more masculine culture. The *Indulgence* vs. *Restraint* dimension correlates positively with boredom scores, indicating lower boredom levels in countries with a more restrained culture. All culture dimensions together explain 1.8% of the variation in epistemic boredom and 2.6% of the variation in activity boredom.

The alternative approach of investigating the culture effect, explaining boredom scores by regions representing different cultures, demonstrates effect sizes in the same order: 2.1% of the variation in epistemic boredom and 2.8% of the variation in activity boredom is explained by regional indicator variables. Against the strict significance requirement applied in this study, only few regions demonstrate statistical significance. Dutch and Belgian students stand out with positive coefficients for the activity boredom explanation. The close proximity of these two regions to the university suggests a difference in selection effects rather than cultural influences causing this effect. This selection effect may also explain the role of the masculinity vs. femininity dimension: in comparison to countries in Germanic Europe and Latin Europe, the Dutch and Belgian regions are characterized by a relatively feminine culture, so the cultural dimension is confounded with the proximity of the country of secondary education.

All together suggest a modest role for cultural dimensions in explaining variation in boredom levels.

### 3.4. Boredom and gender

**Figure 3:**
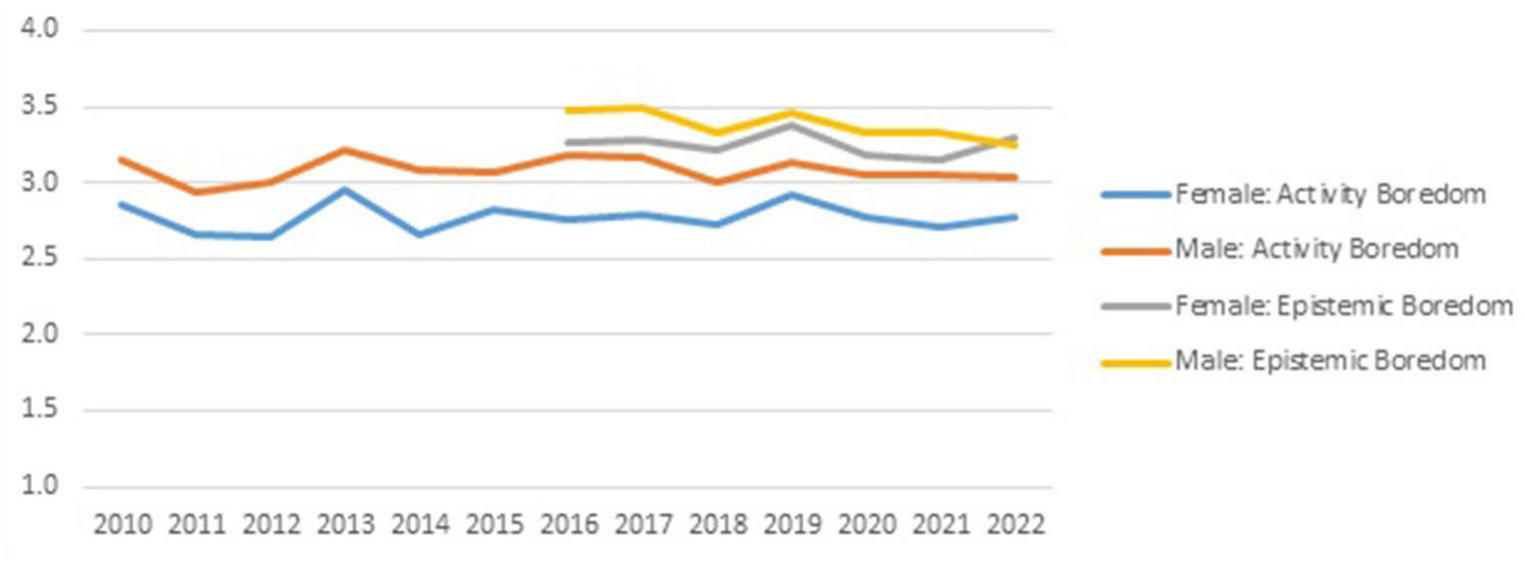
Development of *Activity* and *Epistemic Boredom* over time by gender.

Breaking down the development over time of the activity and epistemic boredom scores, as visible in Figure 2, by gender, gives rise to Figure 3.

A clear gender difference is visible in average boredom levels in all cohorts: average male *Activity Boredom* is 10% higher than average female boredom level, and average male *Epistemic Boredom* level is 5% higher than average female boredom level. However, behind these average levels, there is strong variation in individual boredom scores, implying that gender explains no more than 3.8% of variation *Activity Boredom*, and no more than 1.1% of variation in *Epistemic Boredom*.

### 3.5. Boredom and over- or under-challenge

In discussing the role of being over-challenged, or under-challenged, as an explanation of boredom levels, we apply an objective measure of proficiency: the track of mathematics schooling in high school, expressed by the indicator variable *MathMajor* (1 if the student followed a science preparing mathematical track, 0 if the student followed a social science preparing track). The bivariate correlation of *MathMajor* with *Activity Boredom* equals −0.088, the correlation with *Epistemic Boredom* equals −0.090. Both estimates are statistically significant (*p* < 0.001), but their effect sizes are very small: *MathMajor* explains no more than 0.8% of variation in the two boredom measures. The negative correlations exclude the under-challenge casus; for under-challenge to explain boredom, one would expect positive correlations. Over-challenge is consistent with the negative correlations we observe, but in case learning activities were too challenging, one would have expected both larger effect size and an activity boredom correlation to be more negative than the epistemic boredom correlation. None of these is in full force and effect, making the over- or under-challenge hypothesis not a likely explanation in this context.

### 3.6. Antecedent level differences between *High* and *Low Boredom* profiles

The following steps in the analysis aim to investigate relationships between profile characteristics and potential antecedents. To reduce the impact of *Academic Control* and other factors that may confound the relationship between boredom measures and antecedents, we aggregated the three low boredom profiles into 1 second-order profile *Low Boredom*, and aggregated the three high boredom profiles into 1 second-order profile *High Boredom*. Table 1 summarizes the descriptives of these profiles.

Another advantage of this aggregation of profiles is that this classification is approximately balanced: there is about the same number of high and low boredom students 2,152 vs. 2,443.

**Figure 4:**
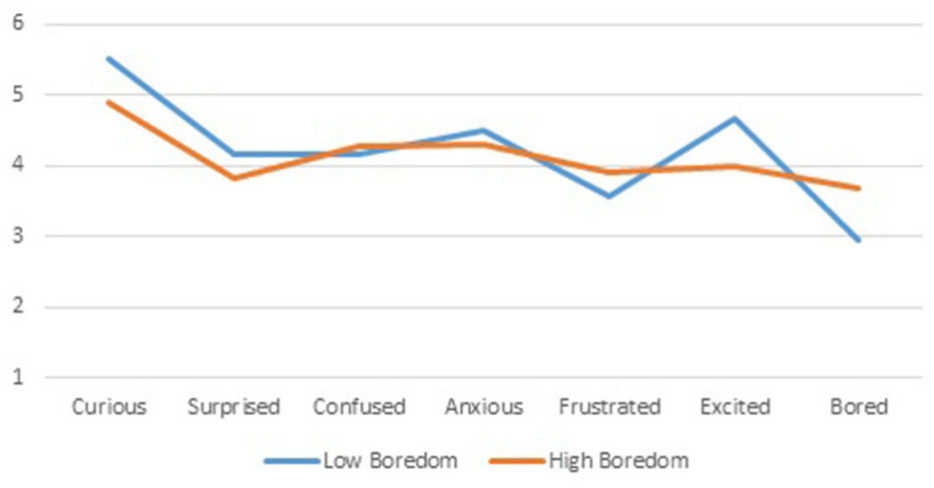
Mean level differences for epistemic emotions of *Low* and *High Boredom* profiles.

In this subsection, we focus on first-order differences between the profiles of students with high activity boredom and low activity boredom: can we observe a difference in mean levels between the profiles? A first candidate for profile differences is epistemic emotions, measured at the very start of the course. Figure 4 provides insight into these level differences.

All level differences in epistemic emotions are statistically significant (beyond the 0.001 level of significance), but only three differences are substantive: *Curious* (eta squared 10.9%), *Excited* (eta squared 8.4%) and *Bored* (eta squared 12.1%). These three epistemic emotions built up during high school education carry over to activity emotions in the current module, whereas the other four epistemic emotions have a less prominent role.

**Figure 5:**
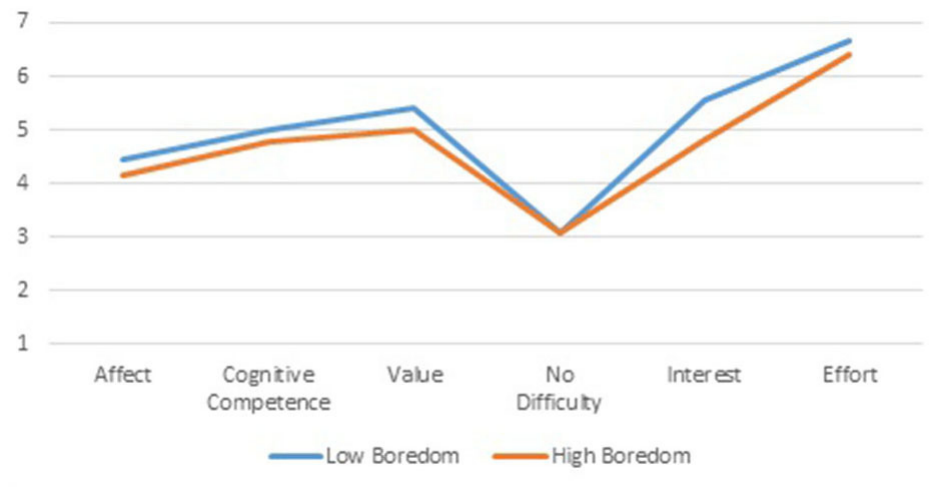
Mean level differences for learning attitudes of *Low* and *High Boredom* profiles.

Learning attitudes based on the expected-value framework count two measures for valuing the learning of quantitative methods: *Value*, representing the extrinsic facet, and *Interest*, representing the intrinsic facet. Profile differences are depicted in Figure 5. All attitudes' scales except students' perception of the difficulty of their learning topics reach statistical significance (*p* < 0.001). However, effect sizes of *Affect* and the expectation component, *Cognitive Competence*, are small: below 2%. The absence of a relationship with perceived difficulty and the small impact of the competence variable contribute to the conclusion that the explanation of boredom out of over-challenge, or under challenge, is highly unlikely. The largest effect sizes refer to the two value components: *Interest*, 11.9%, and *Value*, 5.7%, followed by planned *Effort*, 5.2%.

**Figure 6:**
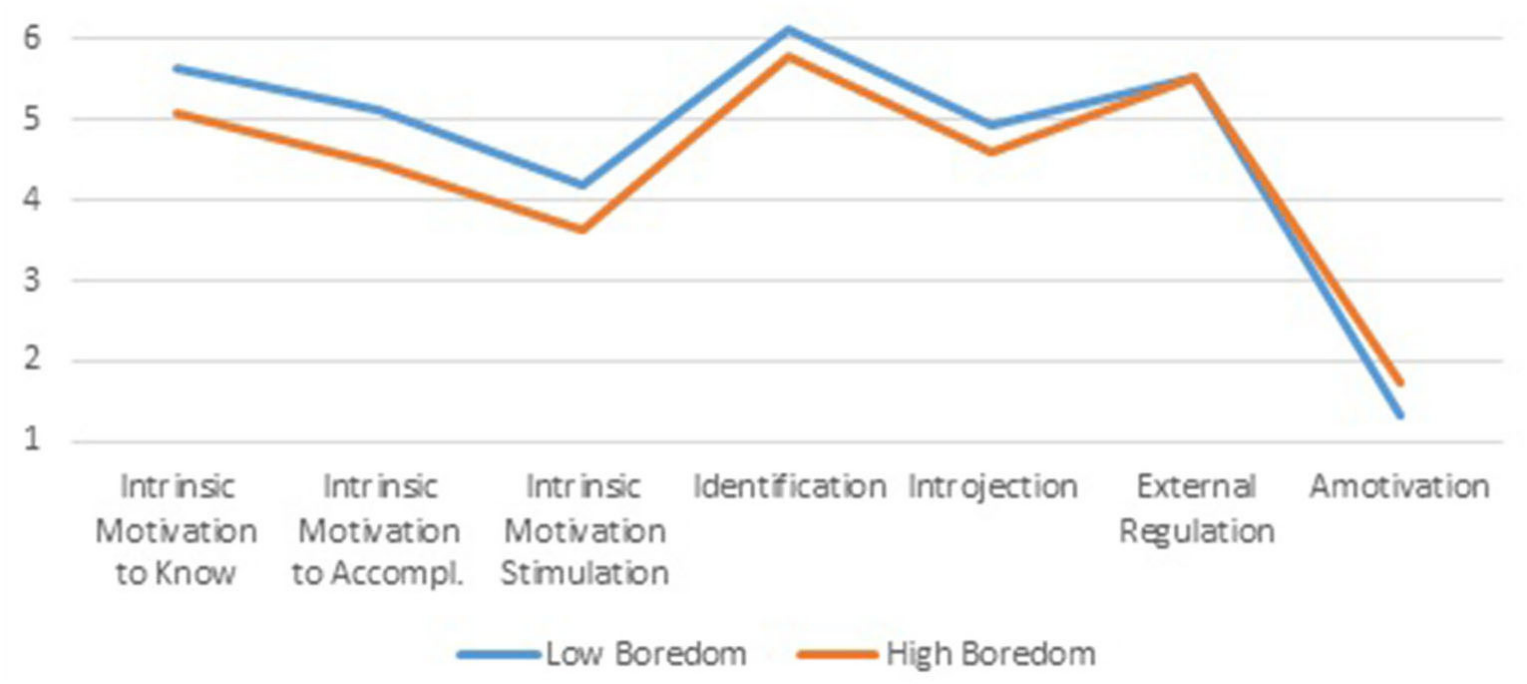
Mean level differences for academic motivation of *Low* and *High Boredom* profiles.

The importance of intrinsic facets of learning motivation in explaining profile differences is also clarified in the Academic Motivation data analysis. Profile differences of three components of intrinsic motivation, on the left-hand side of Figure 6, demonstrate obvious differences in favor of the *Low Boredom* profile. The subsequent three measurements represent motivations that turn from a mixture of intrinsic and extrinsic to purely extrinsic: *External Regulation*. In each step, profile differences diminish (but still stay statistically significant, *p* < 0.001) until they disappear entirely for *External Regulation*. Effect sizes range from 8.8 to 1.7% for *Introjection*.

**Figure 7:**
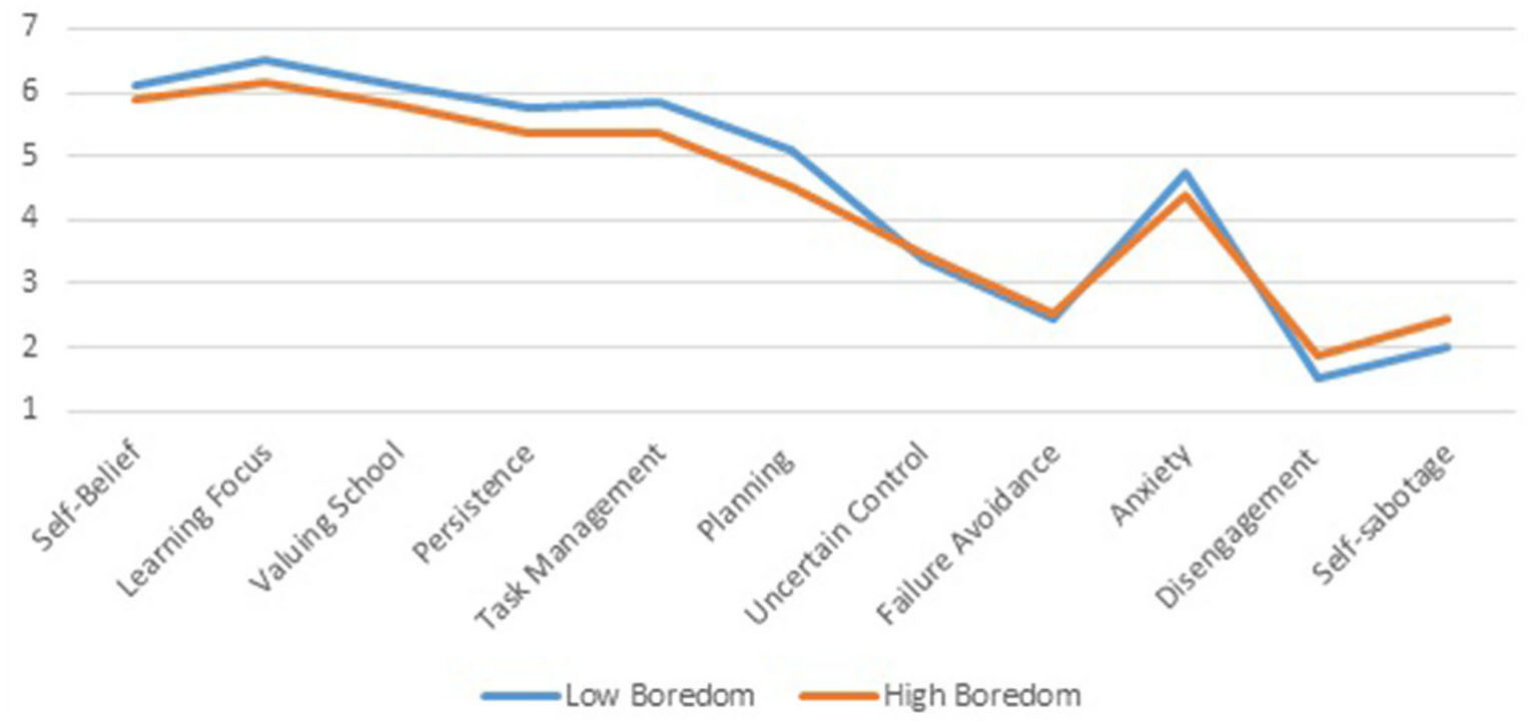
Mean level differences for adaptive and maladaptive motivation and engagement of *Low* and *High Boredom* profiles.

Data from the Motivation & Engagement Wheel help to see another facet of profile differences: the role of adaptive vs. maladaptive learning cognitions and behaviors. In Figure 7, the first three scales refer to adaptive learning cognitions, followed by three adaptive learning behaviors. All profile differences are statistically significant (*p* < 0.001), but the effect of *Self-Belief* lacks substance with an effect size of 1.6% (in line with the small effect size of the learning attitude *Cognitive Competence* discussed above). All other profile differences of adaptive scales are more substantial in size and range between 5.4 and 7.1% effect sizes.

The maladaptive cognitions tell a different story. *Uncertain Control* and *Failure Avoidance* demonstrate a lack of profile differences, whilst *Anxiety* shows a slight profile difference (2.2% effect size) in the opposite direction of other scales. On the other hand, the profile differences in the maladaptive behaviors, *Disengagement* and *Self-sabotage*, are in line with the adaptive measures: significant profile differences with effect sizes of 4.5 and 4.5%.

**Figure 8:**
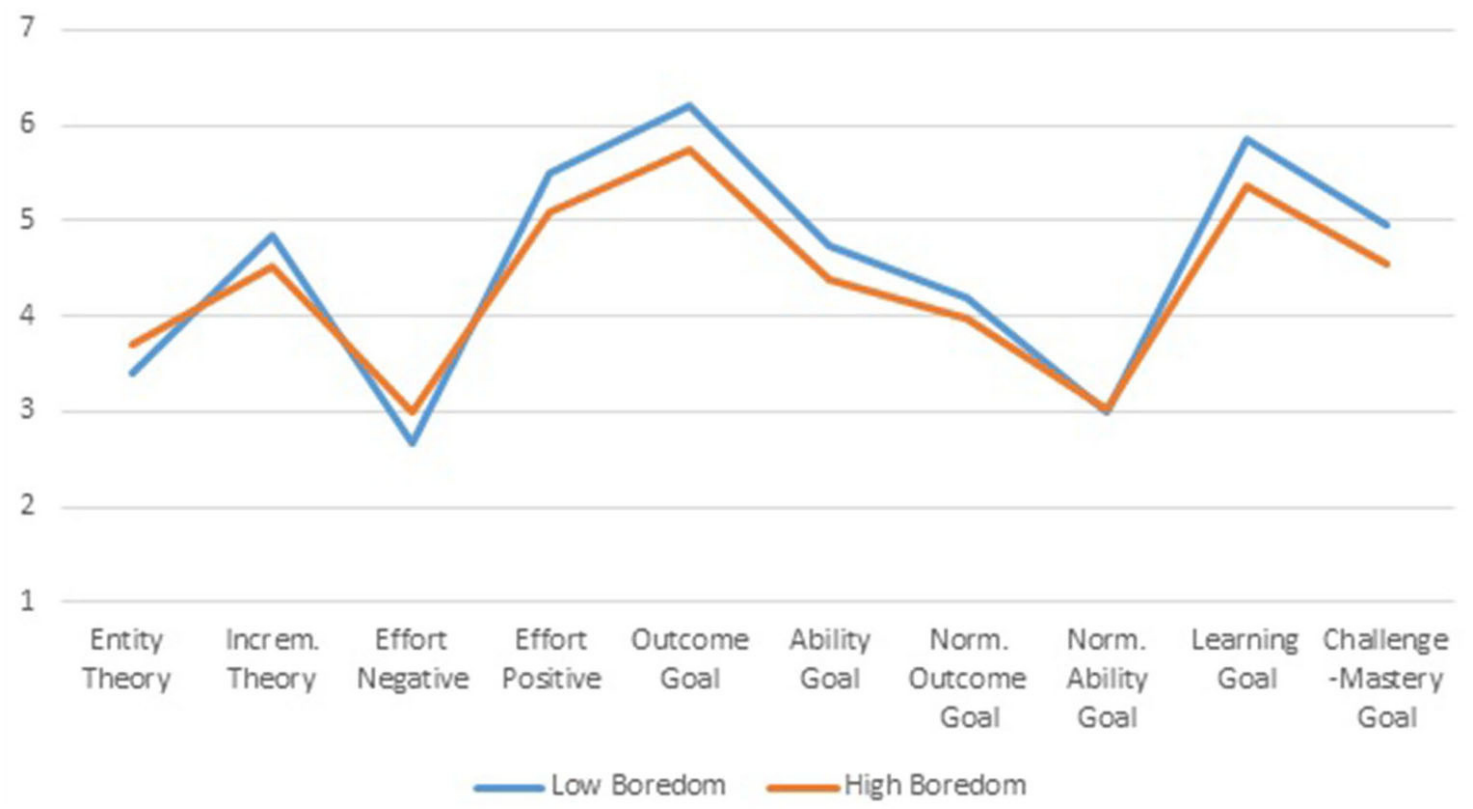
Mean level differences for implicit theories, effort beliefs and goal setting behavior of *Low* and *High Boredom* profiles.

Behind differences in motivational facets, differences in epistemological beliefs that underlie these learning motivations may be expected. Students' mindsets concerning their nature of intelligence and the role of effort in learning constitute one of these beliefs relevant to learning. Profile differences between the two implicit theories of *Entity Theory* and *Incremental Theory*, and the two effort beliefs *Effort Negative* and *Effort Positive*, are displayed in the left part of Figure 8. The right part of Figure 8 displays profile differences in goal setting behavior of the students.

Except for the *Normative Ability Goal*, all profile differences for goal setting are statistically significant (*p* < 0.001). In line with previous research of the author (Tempelaar et al., 2015), we find stronger effects of negative and positive effort beliefs (effect sizes 4.1 and 9.3%, respectively) than for entity and incremental views of intelligence (effect sizes 1.5 and 1.9%, respectively). In addition, somewhat substantial profile differences are present in the two mastery goals *Learning Goal* and *Challenge-Mastery Goal* (effect sizes 9.2 and 4.3%) and the two appearance types of performance goals: *Outcome Goal* and *Ability Goal* (effect sizes 7.7 and 2.3%).

### 3.7. Antecedent relationship differences between *High* and *Low Boredom* profiles

Turning to the last subsection of Results, we focus on second-order differences between the high activity boredom and low activity boredom profiles: can we observe a difference in the relationships of activity boredom and its antecedents, between the profiles of low and high boredom, beyond differences in mean levels. The control-value theory hypothesizes both control and value as antecedents of activity boredom. Control is operationalized as *Academic Control*, and for value, we have two operationalizations available from the learning attitudes instrument: intrinsic value *Interest*, and extrinsic value, *Value*. Next, epistemic boredom acts as the trait-like antecedent of state-like activity boredom. Linear prediction equations based on regression analysis are contained in Table 3, for all students and the two profiles of low and high boredom.

**Table 3:** Prediction equations explaining *Activity Boredom* by *Epistemic Boredom, Academic Control, Interest*, and *Value*, for all students and the two profiles.

	**All**	***Low Boredom***	***High Boredom***
Constant	4.544^***^	3.347^***^	4.960^***^
*Epistemic Boredom*	0.401^***^	0.305^***^	0.318^***^
*Academic Control*	−0.289^***^	−0.206^***^	−0.330^***^
*Interest*	−0.200^***^	−0.059^***^	−0.088^***^
*Value*	−0.068^***^	−0.059^**^	−0.055^*^
R^2^	36.7%	25.1%	26.8%

^*^p < 0.05; ^**^p < 0.01; ^***^p < 0.001.

In the full sample, *Epistemic Boredom* is the dominant predictor of *Activity Boredom*, accounting for more than 60% of explained variation. Within the two profiles, not only explained variation is at a lower level but also the role of *Epistemic Boredom* in that explained variation. Explained variation diminishing with 10% going from the full sample to the two profiles signals that prediction of boredom becomes more difficult after creating homogeneous subsamples. In comparing the two profiles, differences in the regression estimates for Academic Control are prominent. In the *High Boredom* profile, every one-point increase in *Academic Control* comes with a 0.33-point decrease in *Activity Boredom*, on average. In the *Low Boredom* profile, that decrease is no more than 0.21-point, on average.

All the above analyses focus on antecedents of Activity Boredom; in the last paragraphs, we will discuss consequences of boredom. The main consequences all refer to learning performance variables: performance in the final exam and performance in the intermediate quizzes for mathematics and statistics. Bivariate correlations of Activity Boredom with these four consequences for the full sample and the two boredom profiles are displayed in Table 4.

**Table 4:** Correlations of *Activity Boredom* with consequences: the performance measures.

**Activity boredom**	**Mathematics exam**	**Mathematics quizzes**	**Statistics exam**	**Statistics quizzes**
All students	−0.155^***^	−0.151^***^	−0.109^***^	−0.131^***^
*Low Boredom*	−0.117^***^	−0.123^***^	−0.074^***^	−0.133^***^
*High Boredom*	−0.182^***^	−0.124^***^	−0.163^***^	−0.114^***^

^***^p < 0.001.

The major difference between profiles is visible in the correlations with exam score for both topics. Within the *High Boredom* profile, the relationship between exam score and boredom score is much stronger than in the *Low Boredom* profile.

## 4. Conclusions

The most fundamental question in many boredom studies is that what is the best way to measure boredom. This choice of a method though which the concept is operationalized and assessed validly is essential in order to properly capture the experience of boredom. As previously mentioned in the introduction, the observation of boredom presents a challenge in measurement. While qualitative methods such as participant observation (Patton, 2001; Niculescu and Tufanaru, 2005) and in-depth interviews with purposely sampling (Finkielsztein, 2021) are acknowledged as valuable approaches to gather boredom data, they may not be suitable for large student samples in study contexts where researcher observation is not feasible. In such cases, the utilization of quantitative approaches appears more appropriate. Although methodological shortcomings characterize self-report questionnaires, in studying the “silent learning emotion” we may not have a lot of alternatives. Behavioral observation of boredom is difficult, leaving the use of verbal expressions as the most helpful option (Finkielsztein, 2021). Given that “choice from poverty”, it is reassuring that our dispositional learning analytics-based study that applies self-reported emotion measures using verbal expressions generates a consistent set of research outcomes. All relations with boredom antecedents and consequences and relations between epistemic and activity emotions describe a consistent system of learning emotions in which boredom functions as a maladaptive, negatively valenced and deactivating emotion. Although this may prove as a limitation and not demonstrate the validity of self-reported boredom measures, but certainly does not contribute to evidence of the opposite position.

Our study is in some sense “handicapped” by its large sample. Every effect we analyze and every profile difference we investigate is statistically significant, even at the strict requirement of *p* < 0.001. Combining statistical significance with the requirement of an effect size of at least 4%, we conclude that both cultural effects and time effects do not pass this benchmark. The absence of time effects implies that in our data, no effect of the pandemic can be observed. Another “not-in-our-data” phenomenon regards the theory of boredom due to over-challenging or under-challenging learning activities. To demonstrate that phenomenon, we require a significant and substantial relationship between the level of activity boredom and students' prior knowledge, and/or a relationship between activity boredom and the perception of the difficulty of the learning task. None of these can be observed in our data. Given the vast diversity in students' prior schooling, for certain, there have been numerous over-challenged students as well as under-challenged students. However, that cognitive state did not transfer to boredom states. We point to a potential limitation in our study where high stakes for passing this module may have prevented the occurrence of the over- and under-challenge phenomenon.

In line with findings summarized in Finkielsztein (2023) review study, we find gender effects in boredom. Not so much in the frequency of boredom experiences, as reported in Finkielsztein (2023), but in our case in the intensity of boredom. Again, effect sizes are not impressive, but the consistency of male *Activity Boredom* scores exceeding those of female students year after year, with an identical pattern for *Epistemic Boredom*, provides a strong confirmation that previous findings related to the frequency of experiencing boredom, also holds for the intensity of experiencing boredom.

The single situation where statistical significance goes hand in hand with stronger effect sizes is in the difference between *Epistemic* and *Activity Boredom*. On average, the state level of boredom is 11% lower than the trait level of boredom, with an effect size of 31.5%. The trait level of boredom, measured on the threshold between secondary education and university, is the outcome of 6 years of high school mathematics classes. Although the overall mean of trait boredom (3.30) is below the neutral anchor of the scale (4.00), it is remarkable that students experience so much less boredom in doing mathematics and statistics-related learning activities than in their perception of the subjects mathematics and statistics as academic disciplines. Other empirical studies, like Sharp et al. (2021) and Bambrah et al. (2023), also integrate state and trait boredom measures but do not analyze the level differences between the two types of boredom. The question of whether the finding of state-like activity emotions being more positive than corresponding trait-like epistemic emotions can be generalized beyond the situation of this study, remains therefore unanswered. Our results remain however encouraging and should be further validated across different domains and study contexts.

### 4.1. Main contributions and recommendations for further research

The mainstream approach of empirical studies into boredom treats this and other emotions as unitary concepts, in line with the CVTAE model (Pekrun, 2006) and other frameworks. Research by Goetz and co-authors (Nett et al., 2011; Goetz et al., 2014) was the first to point in the direction of a complete typology of boredoms, using experience-sampling methods and frequency measures of boredom. In previous research, Tempelaar and Niculescu (2022), we could generalize the finding of multiple boredom types to a context of self-reported intensity measures of boredom. Most of these boredom types have characteristics that follow from theoretical frameworks such as CVTAE: with higher control comes lower boredom. However, not all types fit in that framework: there exist boredom types where the level of boredom is exceptional relative to arousal or control levels, and relative to levels of other emotions. This study repeats that finding in a slightly different form: we find three profiles of high boredom relative to all other aptitude values, and three levels of low boredom relative to all other aptitude values. That finding is the outcome of creating student profiles by a cluster analysis based on all activity boredom observations. The most notable element of this profiling is maybe not the existence of multiple boredom types in itself, but the outcome that it is only boredom exhibiting multiple types. What makes boredom different from other learning emotions as anxiety and hopelessness, whose behavior is fully aligned with the CVTAE framework? These empirical studies into types of learning emotions suggest that contemporary theoretical frameworks do well in explaining anxiety and hopelessness but repeatedly fall short in explaining boredom (and to a lesser extent: enjoyment). We might need a more advanced theoretical framework for this complex emotion.

Building blocks for such a more advanced theory of learning activity boredom may be found in the reported research findings relating *Activity Boredom* with a broad range of student learning dispositions or aptitudes. Surprisingly, prior knowledge plays a subordinate role, given the lack of a substantial effect of high school mathematics track on boredom, and given the negligible role of cognitive self-perceptions as *Self-Belief* and *Cognitive Competence*. Instead, epistemological beliefs as the intelligence mindsets and related beliefs about the role of effort in learning, non-normative facets of student goal setting, academic motivations at the intrinsic pole and most of the adaptive scales of the Motivation and Engagement Wheel by Martin (2007), candidate for a position in such advanced theoretical framework explaining boredom.

To conclude, the findings from our quantitative, large sample study present boredom as an emotion in the educational setting in a different light than most contemporary theories: boredom is multifaceted and not a single experience in the academic environment. Second, the intensity of this emotion is another aspect to look into beyond how often it is usually reported in current research. Third, we observed minimal time effects in the pandemic and minimal cultural effects. We did notice, however, a gender effect that holds a trend over time for the male students being more bored than their female colleagues are. There are some implications concerning these findings, pointing mainly to the conceptualization of boredom from a theoretical perspective and the need for updated models. Such advancements should also offer more insightful recommendations for specific types of interventions that target the different profiles of boredom exhibited in academic settings and their impact on study retention.

## Data availability statement

The datasets presented in this study can be found via DANS, the Data Archiving and Networked Services of the NOW, the Dutch organization of scientific research. Available online at: https://dans.knaw.nl/en/.

## Ethics statement

The studies involving human participants were reviewed and approved by Ethical Review Committee Inner City faculties (ERCIC) of the Maastricht University. The patients/participants provided their written informed consent to participate in this study.

## Author contributions

DT contributed to the design and implementation of the research, analysis of the results, and writing of the manuscript. AN contributed to the design and implementation of the research and writing of the manuscript. All authors contributed to the article and approved the submitted version.
